# 2-(3-Methyl-2-nitro­phen­yl)-4,5-dihydro-1,3-oxazole

**DOI:** 10.1107/S1600536808040920

**Published:** 2008-12-10

**Authors:** Dongwei Lei, Huibin Yang, Bin Li, Zhuo Kang

**Affiliations:** a Shenyang Institute of Chemical Technology, Shenyang 110142, People’s Republic of China; b Agrochemicals Division, Shenyang Research Institute of Chemical Industry, Shenyang 110021, People’s Republic of China

## Abstract

In the title compound, C_10_H_10_N_2_O_3_, an inter­mediate in the synthesis of anthranilamide insecticides, all the non-H atoms except the nitro-group O atom lie on a crystallographic mirror plane. The H atoms of the methyl group are disordered over two sets of sites with equal occupancies. In the crystal structure, C—H⋯N links lead to chains of mol­ecules propagating in [100].

## Related literature

For background to anthranilamide compounds, a new class of inseticides, see: Lahm *et al.* (2003 ▶, 2005 ▶).
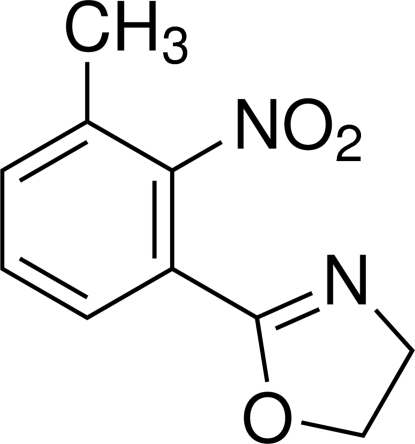


## Experimental

### 

#### Crystal data

C_10_H_10_N_2_O_3_
*M* *_r_* = 206.20
Monoclinic, 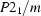

*a* = 7.7767 (10) Å
*b* = 7.3370 (10) Å
*c* = 8.6468 (12) Å
β = 99.414 (2)°
*V* = 486.72 (11) Å^3^
*Z* = 2
Mo *K*α radiation
μ = 0.11 mm^−1^
*T* = 296 (2) K
0.24 × 0.22 × 0.18 mm

#### Data collection

Bruker SMART CCD diffractometer
Absorption correction: multi-scan (*SADABS*; Bruker, 2001 ▶) *T*_min_ = 0.834, *T*_max_ = 1.000 (expected range = 0.818–0.981)
2462 measured reflections
937 independent reflections
842 reflections with *I* > 2σ(*I*)
*R*_int_ = 0.011

#### Refinement

*R*[*F* ^2^ > 2σ(*F* ^2^)] = 0.037
*wR*(*F* ^2^) = 0.110
*S* = 1.07
937 reflections
90 parameters
H-atom parameters constrained
Δρ_max_ = 0.17 e Å^−3^
Δρ_min_ = −0.15 e Å^−3^

### 

Data collection: *SMART* (Bruker, 2005 ▶); cell refinement: *SAINT* (Bruker, 2005 ▶); data reduction: *SAINT*; program(s) used to solve structure: *SHELXS97* (Sheldrick, 2008 ▶); program(s) used to refine structure: *SHELXL97* (Sheldrick, 2008 ▶); molecular graphics: *SHELXTL* (Sheldrick, 2008 ▶); software used to prepare material for publication: *SHELXTL*.

## Supplementary Material

Crystal structure: contains datablocks I, global. DOI: 10.1107/S1600536808040920/hb2868sup1.cif

Structure factors: contains datablocks I. DOI: 10.1107/S1600536808040920/hb2868Isup2.hkl

Additional supplementary materials:  crystallographic information; 3D view; checkCIF report

## Figures and Tables

**Table 1:** Hydrogen-bond geometry (Å, °)

*D*—H⋯*A*	*D*—H	H⋯*A*	*D*⋯*A*	*D*—H⋯*A*
C4—H4⋯N1^i^	0.93	2.60	3.508 (3)	167

Symmetry code: (i) 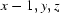.

**Fig. 1.**
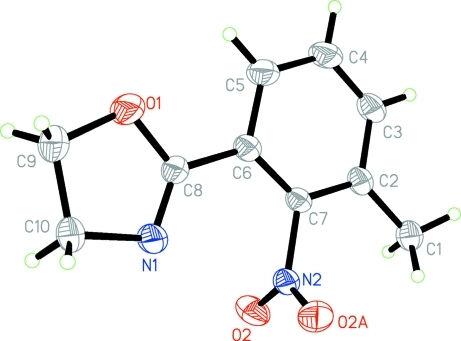
The molecular structure of (I), with 30% probability displacement ellipsoids for the non-hydrogen atoms. Symmetry code: A x, 1/2–y, z.

## supplementary crystallographic information

### Comment

Anthranilamide compounds as a new class of inseticides are characterized by their high levels of insecticidal activity, no-cross resistance to existing insecticides, safety to off-target animal and low toxicity to mammals (Lahm *et al.* 2003, 2005)

The title compound (I) as an intermediate for preparing Chlorantraniliprole analogs plays an important role in identifying the configuration of two possible products.

In the molecular structure of (I), (Fig. 1) all the non-hydrogen atoms except the nitro-group O atom lie on a crystallographic mirror plane. In the crystal, C—H···N links lead to chains of molecules propagating in [100].

### Experimental

2-Bromoethanamine hydrobromide (10.25 g, 50 mmol) and 3-methyl-2-nitrobenzoyl chloride (9.98 g, 50 mmol) were added into dichloromethane (200 ml), then triethylamine (16.70 g, 165 mmol) was added. The mixture was heated to reflux for 14 h and cooled down to room temperature, washed with water and brine, dried by anhydrous sulfate magnesium, then evaporated to give the title compound as a white solid. The product was dissolved in dichloromethane and left to stand at room temperature and colourless blocks of (I) were obtained.

Anal. Calcd for C_10_H_10_N_2_O_3_: C, 58.25; H, 4.89; N, 13.59; O, 23.28. Found: C, 58.20; H, 4.90; N, 13.61; O, 23.25 ^1^H NMR(CDCl_3_): 2.35 (s, 3H, CH_3_), 4.06 (t, J=9.8 Hz, 2H, CH_2_), 4.40 (t, J=9.6 Hz, 2H, CH_2_), 7.41–7.43(m, 2H), 7.78–7.81 (m, 1H).

### Refinement

Although all H atoms were visible in difference maps, they were finally placed in geometrically calculated positions, with C—H distances in the range 0.93–0.96 Å, and included in the final refinement in the riding model approximation, with *U*_iso_(H) = 1.2*U*_eq_(C) for aromatic and *U*_iso_(H) = 1.5*U*_eq_(C) for methyl H atoms.

### Crystal data

**Table:**

C_10_H_10_N_2_O_3_	*F*(000) = 216
*M**_r_* = 206.20	*D*_x_ = 1.407 Mg m^−^^3^
Monoclinic, *P*2_1_/*m*	Mo *K*α radiation, λ = 0.71073 Å
*a* = 7.7767 (10) Å	Cell parameters from 1713 reflections
*b* = 7.337 (1) Å	θ = 2.7–27.9°
*c* = 8.6468 (12) Å	µ = 0.11 mm^−^^1^
β = 99.414 (2)°	*T* = 296 K
*V* = 486.72 (11) Å^3^	BLOCK, colourless
*Z* = 2	0.24 × 0.22 × 0.18 mm

### Data collection

**Table:**

Bruker SMART CCD diffractometer	937 independent reflections
Radiation source: fine-focus sealed tube	842 reflections with *I* > 2σ(*I*)
graphite	*R*_int_ = 0.011
ω scans	θ_max_ = 25.0°, θ_min_ = 2.4°
Absorption correction: multi-scan (*SADABS*; Bruker, 2001)	*h* = −9→9
*T*_min_ = 0.834, *T*_max_ = 1.000	*k* = −6→8
2462 measured reflections	*l* = −10→9

### Refinement

**Table:**

Refinement on *F*^2^	Primary atom site location: structure-invariant direct methods
Least-squares matrix: full	Secondary atom site location: difference Fourier map
*R*[*F*^2^ > 2σ(*F*^2^)] = 0.037	Hydrogen site location: inferred from neighbouring sites
*wR*(*F*^2^) = 0.110	H-atom parameters constrained
*S* = 1.07	*w* = 1/[σ^2^(*F*_o_^2^) + (0.0583*P*)^2^ + 0.1052*P*] where *P* = (*F*_o_^2^ + 2*F*_c_^2^)/3
937 reflections	(Δ/σ)_max_ < 0.001
90 parameters	Δρ_max_ = 0.17 e Å^−^^3^
0 restraints	Δρ_min_ = −0.15 e Å^−^^3^

### Special details

**Table:**

Geometry. All e.s.d.'s (except the e.s.d. in the dihedral angle between two l.s. planes) are estimated using the full covariance matrix. The cell e.s.d.'s are taken into account individually in the estimation of e.s.d.'s in distances, angles and torsion angles; correlations between e.s.d.'s in cell parameters are only used when they are defined by crystal symmetry. An approximate (isotropic) treatment of cell e.s.d.'s is used for estimating e.s.d.'s involving l.s. planes.
Refinement. Refinement of *F*^2^ against ALL reflections. The weighted *R*-factor *wR* and goodness of fit *S* are based on *F*^2^, conventional *R*-factors *R* are based on *F*, with *F* set to zero for negative *F*^2^. The threshold expression of *F*^2^ > σ(*F*^2^) is used only for calculating *R*-factors(gt) *etc*. and is not relevant to the choice of reflections for refinement. *R*-factors based on *F*^2^ are statistically about twice as large as those based on *F*, and *R*- factors based on ALL data will be even larger.

### Fractional atomic coordinates and isotropic or equivalent isotropic displacement parameters (Å^2^)

**Table:**

	*x*	*y*	*z*	*U*_iso_*/*U*_eq_	Occ. (<1)
O1	0.2190 (2)	0.2500	1.12373 (17)	0.0794 (6)	
O2	0.33918 (12)	0.10351 (17)	0.61731 (13)	0.0628 (4)	
N1	0.4057 (2)	0.2500	0.95534 (19)	0.0617 (6)	
N2	0.27535 (19)	0.2500	0.64083 (17)	0.0428 (4)	
C1	−0.0249 (3)	0.2500	0.4054 (2)	0.0520 (5)	
H1A	0.0705	0.3268	0.3893	0.078*	0.50
H1B	−0.1309	0.2952	0.3449	0.078*	0.50
H1C	−0.0041	0.1280	0.3728	0.078*	0.50
C2	−0.0410 (2)	0.2500	0.5759 (2)	0.0430 (5)	
C3	−0.2030 (3)	0.2500	0.6245 (3)	0.0533 (5)	
H3	−0.3034	0.2500	0.5495	0.064*	
C4	−0.2181 (3)	0.2500	0.7801 (3)	0.0630 (6)	
H4	−0.3281	0.2500	0.8092	0.076*	
C5	−0.0709 (3)	0.2500	0.8946 (3)	0.0589 (6)	
H5	−0.0828	0.2500	0.9998	0.071*	
C6	0.0946 (2)	0.2500	0.8531 (2)	0.0434 (5)	
C7	0.1040 (2)	0.2500	0.6938 (2)	0.0381 (4)	
C8	0.2508 (2)	0.2500	0.9762 (2)	0.0428 (5)	
C9	0.3878 (3)	0.2500	1.2242 (3)	0.0671 (7)	
H9	0.4021	0.1424	1.2902	0.081*	
C10	0.5156 (3)	0.2500	1.1111 (2)	0.0580 (6)	
H10	0.5890	0.3576	1.1251	0.070*	

### Atomic displacement parameters (Å^2^)

**Table:**

	*U*^11^	*U*^22^	*U*^33^	*U*^12^	*U*^13^	*U*^23^
O1	0.0487 (9)	0.1510 (17)	0.0384 (8)	0.000	0.0066 (7)	0.000
O2	0.0475 (6)	0.0714 (8)	0.0701 (8)	0.0137 (5)	0.0117 (5)	−0.0128 (5)
N1	0.0353 (9)	0.1100 (16)	0.0376 (9)	0.000	−0.0005 (7)	0.000
N2	0.0323 (8)	0.0582 (10)	0.0363 (8)	0.000	0.0013 (6)	0.000
C1	0.0451 (11)	0.0637 (13)	0.0437 (11)	0.000	−0.0033 (8)	0.000
C2	0.0358 (10)	0.0443 (10)	0.0463 (10)	0.000	−0.0010 (8)	0.000
C3	0.0316 (9)	0.0658 (13)	0.0594 (13)	0.000	−0.0020 (8)	0.000
C4	0.0322 (10)	0.0932 (17)	0.0649 (14)	0.000	0.0114 (9)	0.000
C5	0.0397 (11)	0.0885 (16)	0.0505 (12)	0.000	0.0130 (9)	0.000
C6	0.0347 (10)	0.0516 (11)	0.0434 (10)	0.000	0.0046 (8)	0.000
C7	0.0290 (8)	0.0422 (10)	0.0428 (10)	0.000	0.0047 (7)	0.000
C8	0.0408 (10)	0.0521 (11)	0.0352 (9)	0.000	0.0057 (7)	0.000
C9	0.0550 (13)	0.1013 (19)	0.0413 (11)	0.000	−0.0030 (10)	0.000
C10	0.0445 (11)	0.0834 (16)	0.0418 (11)	0.000	−0.0058 (8)	0.000

### Geometric parameters (Å, °)

**Table:**

O1—C8	1.339 (2)	C3—C4	1.370 (3)
O1—C9	1.451 (3)	C3—H3	0.9300
O2—N2	1.2149 (13)	C4—C5	1.385 (3)
N1—C8	1.247 (2)	C4—H4	0.9300
N1—C10	1.472 (2)	C5—C6	1.391 (3)
N2—O2^i^	1.2149 (13)	C5—H5	0.9300
N2—C7	1.478 (2)	C6—C7	1.391 (3)
C1—C2	1.501 (3)	C6—C8	1.478 (3)
C1—H1A	0.9600	C9—C10	1.504 (3)
C1—H1B	0.9600	C9—H9	0.9700
C1—H1C	0.9600	C9—H9^i^	0.9700
C2—C7	1.391 (2)	C10—H10	0.9700
C2—C3	1.391 (3)	C10—H10^i^	0.9700
			
C8—O1—C9	106.31 (16)	C6—C5—H5	119.8
C8—N1—C10	107.34 (17)	C7—C6—C5	117.07 (18)
O2^i^—N2—O2	124.43 (16)	C7—C6—C8	122.89 (17)
O2^i^—N2—C7	117.75 (8)	C5—C6—C8	120.04 (18)
O2—N2—C7	117.75 (8)	C2—C7—C6	123.93 (17)
C2—C1—H1A	109.5	C2—C7—N2	115.90 (16)
C2—C1—H1B	109.5	C6—C7—N2	120.18 (15)
H1A—C1—H1B	109.5	N1—C8—O1	118.10 (17)
C2—C1—H1C	109.5	N1—C8—C6	126.55 (17)
H1A—C1—H1C	109.5	O1—C8—C6	115.35 (16)
H1B—C1—H1C	109.5	O1—C9—C10	103.87 (16)
C7—C2—C3	116.38 (18)	O1—C9—H9	111.0
C7—C2—C1	122.16 (17)	C10—C9—H9^i^	111.0
C3—C2—C1	121.46 (17)	O1—C9—H9^i^	111.0
C4—C3—C2	121.60 (18)	C10—C9—H9^i^	111.0
C4—C3—H3	119.2	H9—C9—H9^i^	109.0
C2—C3—H3	119.2	N1—C10—C9	104.38 (16)
C3—C4—C5	120.52 (19)	N1—C10—H10	110.9
C3—C4—H4	119.7	C9—C10—H10	110.9
C5—C4—H4	119.7	N1—C10—H10^i^	110.9
C4—C5—C6	120.5 (2)	C9—C10—H10^i^	110.9
C4—C5—H5	119.8	H10—C10—H10^i^	108.9
			
C7—C2—C3—C4	0.0	O2—N2—C7—C2	−88.46 (13)
C1—C2—C3—C4	180.0	O2^i^—N2—C7—C6	−91.54 (13)
C2—C3—C4—C5	0.0	O2—N2—C7—C6	91.54 (13)
C3—C4—C5—C6	0.0	C10—N1—C8—O1	0.0
C4—C5—C6—C7	0.0	C10—N1—C8—C6	180.0
C4—C5—C6—C8	180.0	C9—O1—C8—N1	0.0
C3—C2—C7—C6	0.0	C9—O1—C8—C6	180.0
C1—C2—C7—C6	180.0	C7—C6—C8—N1	0.0
C3—C2—C7—N2	180.0	C5—C6—C8—N1	180.0
C1—C2—C7—N2	0.0	C7—C6—C8—O1	180.0
C5—C6—C7—C2	0.0	C5—C6—C8—O1	0.0
C8—C6—C7—C2	180.0	C8—O1—C9—C10	0.0
C5—C6—C7—N2	180.0	C8—N1—C10—C9	0.0
C8—C6—C7—N2	0.0	O1—C9—C10—N1	0.0
O2^i^—N2—C7—C2	88.46 (13)		

Symmetry codes: (i) *x*, −*y*+1/2, *z*.

### Hydrogen-bond geometry (Å, °)

**Table:**

*D*—H···*A*	*D*—H	H···*A*	*D*···*A*	*D*—H···*A*
C4—H4···N1^ii^	0.93	2.60	3.508 (3)	167

Symmetry codes: (ii) *x*−1, *y*, *z*.
